# Systematic review of complications with externally controlled motorized intramedullary bone lengthening nails (FITBONE and PRECICE) in 983 segments

**DOI:** 10.1080/17453674.2020.1835321

**Published:** 2020-10-27

**Authors:** Markus W Frost, Ole Rahbek, Jens Traerup, Adriano A Ceccotti, Søren Kold

**Affiliations:** a Department of Orthopedic Surgery, Aalborg University Hospital, Aalborg;; b Department of Clinical Medicine, Faculty of Medicine, Aalborg University, Aalborg, Denmark

## Abstract

Background and purpose — In recent years motorized intramedullary lengthening nails have become increasingly popular. Complications are heterogeneously reported in small case series and therefore we made a systematic review of complications occurring in lower limb lengthening with externally controlled motorized intramedullary bone lengthening nails.

Methods — We performed a systematic search in PubMed, EMBASE, and the Cochrane Library with medical subject headings: Bone Nails, Bone Lengthening, and PRECICE and FITBONE nails. Complications were graded on severity and origin.

Results — The search identified 952 articles; 116 were full text screened, and 41 were included in the final analysis. 983 segments were lengthened in 782 patients (age 8–74 years). The distribution of nails was: 214 FITBONE, 747 PRECICE, 22 either FITBONE or PRECICE. Indications for lengthening were: 208 congenital shortening, 305 acquired limb shortening, 111 short stature, 158 with unidentified etiology. We identified 332 complications (34% of segments): Type I (minimal intervention) in 11% of segments; Type II (substantial change in treatment plan) in 15% of segments; Type IIIA (failure to achieve goal) in 5% of segments; and Type IIIB (new pathology or permanent sequelae) in 3% of segments. Device and bone complications were the most frequent.

Interpretation — The overall risk of complications was 1 complication for every 3 segments lengthened. In 1 of every 4 segments, complications had a major impact leading to substantial change in treatment, failure to achieve lengthening goal, introduction of a new pathology, or permanent sequelae. However, as no standardized reporting method for complications exists, the true complication rates might be different.

Distraction osteogenesis through an externally applied fixator is a well-established treatment for lower limb lengthening (De Bastiani et al. 1987, Paley 1988, Ilizarov 1990). However, complication rates of this treatment are high, amounting to 1–3.2 complications per patient (Tjernström et al. 1994, Noonan et al. 1998). The wires or pins penetrating soft tissues result in complications such as pin site infection, pain, scarring, muscle transfixation, reduced joint movement, and immobility (Paley 1990, Mazeau et al. 2012, Landge et al. 2015). When the external fixator is removed, there is a risk of further complications such as fracture or malalignment (Noonan et al. 1998, Simpson and Kenwright 2000). To reduce complications and improve patient comfort, limb lengthening by fully implantable bone lengthening nails has been introduced (Guichet 1999, Cole et al. 2001). Problems with purely mechanically driven lengthening nails were resolved by the introduction of motorized (FITBONE) or magnetically driven (PRECICE) bone lengthening nails (Baumgart et al. 1997, Kirane et al. 2014, Paley et al. 2014, Shabtai et al. 2014). A few case-control studies have compared these nails with external fixation (13–15 patients), and the largest case series on intramedullary bone lengthening reports on 92 patients (Black et al. 2015, Horn et al. 2015, Calder et al. 2019). However, the majority of reports of complications of the FITBONE and PRECICE lengthening nails are small case series (Krieg et al. 2008, Dinçyürek et al. 2012, Birkholtz and De-Lange 2016, Hammouda et al. 2017). In recent years motorized intramedullary lengthening nails have become increasingly popular, and we thus hypothesized that standardized data on complications could now be extracted from the literature. We performed a systematic literature review of complications using PRECICE and FITBONE bone lengthening nails in lower limb bone lengthening. The primary outcome was risk of complications imposing a new pathology or permanent sequelae in the patient.

## Method

### Search criteria

An electronic search in the databases PubMed, Embase, and Cochrane Library was performed by a health science librarian with expertise in systematic literature searching. For details of the search strategy see Supplementary data 1. There was no limit concerning study design, publishing date, or language. We searched reference lists of included studies, relevant reviews identified through the systematic search and authors’ personal files to ensure literature saturation.

### Inclusion and exclusion criteria

We included only published full-text original studies designed as randomized controlled trials, prospective and retrospective cohort studies, case-control studies, case series, and case reports. Cross-sectional studies were excluded. Studies in both English and German were included.

Studies were included if: the bone lengthening nails applied were FITBONE (Wittenstein Intens GmbH, Igersheim, Germany) and/or PRECICE (Nuvasive, San Diego, CA, USA), conducted in humans, and bone lengthening was performed on lower extremities. Descriptions of complications included origin, severity, and management of complications or a statement of no complications. Studies were excluded if: reporting only bone transport treatment, nails were used only for compression, there was no involvement of lower extremities, or reporting stump lengthening. If patients were represented in more than one study, only one of the studies was included. A single patient or a group of patients from one study could be included if patient-/group-specific data was available.

### Data collection and management

The primary search was performed at the end of November 2019 and updated at the end of March 2020. The literature search was assembled in www.covidence.org as well as the management of article selection flow. Titles and abstracts were screened by the first author (MWF) to select articles for full text reading. Among the full text articles, MWF selected papers for possible inclusion. SK and MWF assessed articles in accordance with inclusion criteria and agreed on studies relevant for final inclusion.

During the initial data collection, MWF collected the following information from each study: title, author(s), year of publication, study design, evidence level, number of patients, number of lengthening segments, sex, nail type (FITBONE or PRECICE), participant age range, and bone segments (femur or tibia). Etiology was divided into 3 groups: (1) congenital, (2) short stature, (3) acquired/developed limb length discrepancy diagnoses in accordance with the modified Stricker and Hunt classification (Stricker and Hunt 2004) (see Supplementary data 2), min./max. leg length, and perioperative soft tissue release. Complications were assessed according to the particular point in time when they occurred: intraoperative complication (Early 1:E1), postoperative complication prior to distraction start (Early 2:E2), during distraction period (Late 1:L1), after end of distraction and prior to implant removal (Late 2:L2), and after implant removal (Late 3:L3) (see Supplementary data 3). The severity of complications was classified according to Black et al. (2015) (Table 1). If a complication was graded according to Paley, we used Table 1 to compile the complication into the Black classification. If the treatment of a complication was not thoroughly described, we generally downgraded it, assuming that the treatment goal was achieved and no new pathology or permanent sequalae had emerged. As an example, a joint contracture with no described changes in treatment was classified as grade I. If a complication was graded by article authors as grade I, but the described treatment included additional surgery, we graded it as grade II. A deep vein thrombosis was graded as grade IIIB. The type of complication was categorized into origin representing 8 main groups (soft tissue, joint, vascular, bone, neurological, infection, device-related, others) and 33 subgroups according to Table 5 (for specific examples see Supplementary data 4). Intra-articular nail placement causing irritation and residual deformity was categorized into origin as Others/Surgical. Patient requesting to stop the lengthening procedure was categorized into origin as Others/Patient.

**Table 1.** Classification of severity of complications in accordance with Black et al. and Paley

Complication severity grade			Examples of complications	
Modified Black et al. 2015	Paley 1990			
I	Minimal intervention required; treatment goal still achieved	Problems	Potential expected difficulty arising during distraction or fixation period which is fully resolved non-operatively by end of the treatment period	Pin-site infection. Temporary joint contracture
II	Substantial change in treatment plan; treatment goal still achieved	Obstacle	Potential expected difficulty that arose doing distraction or fixation period that is fully resolved by end of the treatment period by operative means	Unplanned return to surgery, such as delayed consolidation requiring additional intervention, and device problem needing revision
IIIA	Failure to achieve treatment goal; no new pathology or permanent sequelae. Peri- or intraoperative complication without sequelae	Complication	Complication include any local or systemic intraoperative or perioperative complication, difficulty during distraction or fixation that remains unsolved at the end of treatment period, and any early or late post-treatment difficulty	Premature consolidation with aborted lengthening, inability to tolerate lengthening, and fracture at fixation site or regenerate bone with shortening
IIIB	Failure to achieve treatment goal and/or new pathology or permanent sequelae		Complications were divided into minor and major depending on whether the original treatment goal was achieved	Joint subluxation, joint dislocation, regenerate fracture with deformity, and deep infection. Thromboembolic complication such as deep vein thrombosis

Flow diagram of selection of studies.

**Table 5.** Complications categorized into 8 main groups (soft tissue, joint, vascular, bone, neurological, infection, device-related, others) and 33 subgroups

	Severity grade and origin of complications				
Group	I	II	IIIA	IIIB	Sum
Soft tissue					
Skin	2	1			3
Muscles					0
Tendons					0
Pain	5				5
Others	2	1		2 (CS)	5
Sum of soft tissue					13
Soft tissue complications in % of segments			1		
Joint					
Pain	1				1
Contracture	19	24	5	5	53
Subluxation				6	6
Dislocation				1	1
Others					0
Sum of joint					61
Joint complications % of segment:				6	
Vascular					
Vascular damage				1	1
Deep vein thrombosis				4	4
Hemorrhage/hematoma	2				2
Others	2			1 (AV)	3
Sum of vascular					10
Vascular complications in % of segments:				1	
Bone					
Premature consolidation		15	4		19
Delayed healing	16	27	2	1	46
Secondary malalignment		1		2	3
Fracture		6	1	1	8
Others	1	1			2
Sum of bone					78
Bone complications in % of segments:				8	
Neurology					
Paresthesia	2	1	2		5
Paralysis					0
Others	3				3
Sum of neurology					8
Neurology complication in % of segments:				0.8	
Infection					
Superficial soft tissue	2	1			3
Deep soft tissue		1			1
Osteomyelitis			3	1	4
Others					0
Sum of infection					8
Infection complications in % of segments:				0.8	
Device-related					
Distraction mechanism	16	20	9		45
Mechanical strength	25	14	3	2	44
Attachment failure	8	24	1		33
Others					0
Sum of device-related					122
Device-related complications in % of segments:			12		
Others					
Patient			6		6
Surgical		3	7	1	11
Others			1		1
Sum of others					18
Others, complications in % of segments				1.8	

CS: compartment syndrome;

AV: arteriovenous fistula of the posterior tibial artery decompensated during tibial lengthening and an embolization procedure had to be performed. 14 complications could not be categorized due to missing descriptions.

MWF identified all complications and graded them according to severity, time of treatment, and origin. A second reviewer (SK) subsequently evaluated and graded the complication concerning severity and origin. Disagreement between reviewers was solved by consensus discussion.

The Oxford Centre for Evidence-Based Medicine—Levels of Evidence 2009 grading of Harm was used to assess the level of evidence in the included studies (case reports were not included). A study was classified as a case report if reporting less than 5 bone lengthening segments. Case series with a subgroup analysis were classified as a cohort study. A study was considered prospective if data were collected prospectively; all other studies were considered retrospective.

We used a methodology quality assessment score for all studies: Methodological Index for Non-Randomized Studies (MINORS) for non-randomized studies; Murad et al. for case reports (Slim et al. 2003, Murad et al. 2018). MWF and AG independently assessed the studies and solved difference through discussion. 3 specific questions concerning harms (from the McHarm scale) were used (Santaguida et al. 2011, Kronick et al. 2014) (see methodology quality assessment score, Supplementary data 5).

### Statistics

Microsoft Excel 2019 version 16.33 (Microsoft Corp, Redmond, WA, USA) was used for data storage and descriptive analysis. Inter-rater agreement between the 2 assessors of complications was calculated as Kappa values for both severity grading of complications (4 types) and categorization of origin (8 main groups and 33 sub-groups) with Stata/MP 15.1 (StataCorp, College Station, TX, USA). For the strength of agreement, values less than 0 were rated as Poor; 0–0.20 Slight; 0.21–0.40 Fair; 0.41–0.60 Moderate; 0.61–0.8 Substantial; and 0.80–1 Almost perfect (Landis and Koch 1977).

**Figure:**
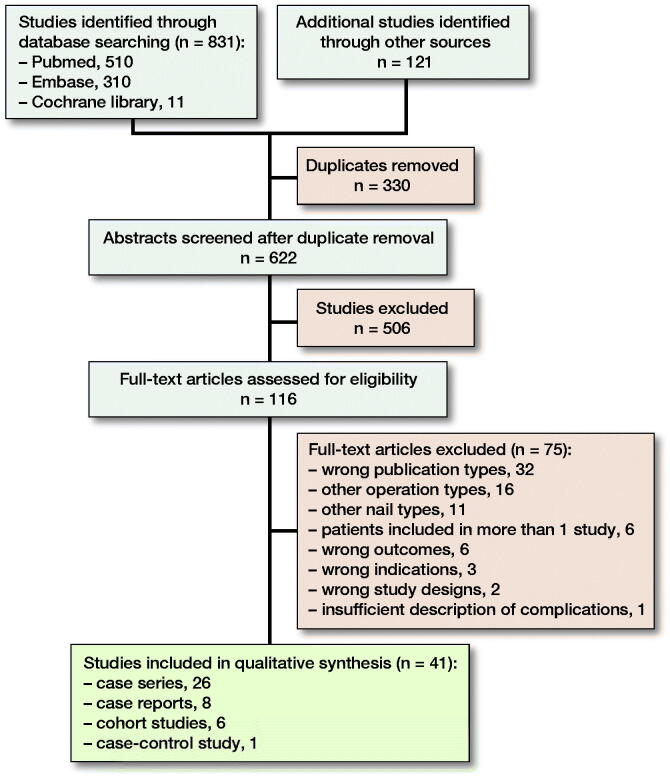
Flow diagram of selection of studies.

### Registration, funding, and potential conflicts of interest

Prior to conducting the study, we searched the PROSPERO database (http://www.crd.york.ac.uk/PROSPERO) for ongoing reviews and recently completed systematic reviews; we did not identify any results. This study was then submitted to PROSPERO on November 22, 2019. Due to waiting time at PROSPERO, this pre-study registration has unfortunately not been published before submission (April 23, 2020). During the review period, the pre-study registration was published by PROSPERO (ID number: CRD42020159272). The majority of studies included were case series and case reports and we changed the risk of bias/quality assessment tool to MINORS and added Murad et al. for case reports (Slim et al. 2003, Murad et al. 2018). Thus, reporting guidelines were also changed to Meta-analysis of Observational Studies in Epidemiology (MOOSE), and this systematic review was organized in agreement with this (Stroup et al. 2000). The change was submitted to PROSPERO. The authors’ institutions funded the study. No conflicts of interest are declared.

## Results

Our search identified 952 articles of which 41 were included (Table 2); for flowchart of article selection see Figure. There was 1 case-control study, 6 cohort studies, 26 case series, and 8 case reports. Of the 33 studies that were not case reports, there was 1 level 3 study and 32 level 4 studies. The mean MINORS score was 8.3 (n = 26, range 5–12, ideal score 16) for non-comparative studies and 15.1 (n = 7, range 12–18, ideal score 24) for comparative studies (for full score of studies, see Supplementary data 6). The mean score for case reports was 4.3 (n = 8, 3–6 range, ideal score 8). Concerning the McHarm questions: (1) 1 study included predefined/standardized descriptions of complications, (2) standard scale of complications was used in 15 studies, and (3) number of each type of event and total number were specified on study groups in 31 studies (see Supplementary data 6).

**Table 2.** Included studies with the corresponding number of patients, segments, and complications used in this review

Reference	Prospective	Patients n	Segments n	Nail type a	Complications
Accadbled et al. 2019	Yes	5	7	F	1
Accadbled et al. 2016	Yes	23	26	F	9
Al-Sayyad 2012	Yes	10	14	F	1
Baumgart et al. 1997	No	11	11	F	6
Baumgart et al. 2005	No	1	3	F	0
Birkholtz and De-Lange 2016	No	9	11	P	2
Black et al. 2015	No	13	15	F	20
Calder et al. 2019	No	92	107	P	31
Cosic and Edwards 2020	No	21	21	P	9
Couto et al. 2018	No	1	2	P	0
Dinзyьrek et al. 2012	No	14	15	F	12
Frommer et al. 2018	No	54	60	P	7
Haider and Wozasek 2019	No	20	20	P	8
Hammouda et al. 2017	No	17	17	P	4
Harkin et al. 2018	No	3	3	P	0
Havitcioglu et al. 2020	No	8	16	P/ F	4
Horn et al. 2019	No	47	50	P/ F	16
Iobst et al. 2018	No	27	27	P	4
Karakoyun et al. 2016	No	23	27	P	10
Karakoyun et al. 2015	No	22	22	F/P	2
Kariksiz and Karakoyun 2019	No	1	1	P	0
Kirane et al. 2014	No	24	25	P	6
Krieg et al. 2008	Yes	8	8	F	4
Krieg et al. 2011	No	32	32	F	10
Kьзьkkaya et al. 2015	No	22	25	F	5
Laubscher et al. 2016	No	15	20	P	5
Lee et al. 2017	No	41	80	P	36
Lenze et al. 2011	No	11	11	F	6
Morrison and Sontich 2016	No	1	1	P	1
Muratori et al. 2018	No	4	4	P	1
Nasto et al. 2020	Yes	26	26	P	10
Paley et al. 2015	No	51	116	P	20
Paley et al. 2014	No	46	62	P	31
Rozbruch 2017	No	2	2	P	1
Schiedel et al. 2014	Yes	24	26	P	9
Shabtai et al. 2014	Yes	18	21	P	9
Singh et al. 2006	No	10	24	F	14
Steiger et al. 2018	No	5	5	F	2
Tiefenboeck et al. 2016	No	10	10	P	13
Wiebking et al. 2016	No	9	9	P	2
Wu and Kuhn 2018	No	1	1	P	1

a F = FITBONE; P = PRECICE.

The 41 studies included 782 patients and 983 bone lengthening segments (Table 3). We found 332 complications corresponding to 34% of segments; 14 complications were not classified with origin, only severity. We observed 28 type IIIB complications, which was our primary outcome, corresponding to 3% of segments. Type IIIA complications not achieving the lengthening goal were seen in 45 cases (5% of segments). There were 113 type I complications and 146 type II complications, corresponding to 11% and 15% complications per segment, respectively (Table 4). Device-related complications (12% of segments) were the most frequent type of complication followed by bone (8% of segments) and then joint complications (6% of segments) (Table 5).

**Table 4.** Severity grading of complications divided into specific numbers and percentages of lengthened segments and patients

	Severity grade of complications				
Factor	I	II	IIIA	IIIB	Sum
Number of complications	113	146	45	28	332
Complications per segment, %	11	15	5	3	34
Complications per patient, %	14	19	6	4	42

Grading according to severity by Black et al. (2015).

**Table 3.** Descriptive study data collected from the studies reporting group-level data

Factor	Numbers	Studies reporting data
Number of patients	782	41
Number of bone segments	983	41
Male / female, n	384 / 234	29 / 33
Age, min / max	8 / 74	39
Etiology, n		
Congenital disease	208	22
Short stature	111	14
Acquired/developmental LLD	305	29
Femur / tibia, n	813 / 170	40 / 28
Bone lengthening cm, min / max	1 / 14	35 / 35
FITBONE/ PRECICE nails	214 / 747	15 / 27

164 patients were unidentified regarding gender, and 158 patients regarding etiology.

22 nails could not be differentiated between FITBONE or PRECICE. LLD: limb length discrepancy

5 studies reported a systematic approach to soft-tissue release during primary surgery (Shabtai et al. 2014, Paley et al. 2015, Laubscher et al. 2016, Rozbruch 2017, Calder et al. 2019). None of the 41 studies systematically reported the timing of the complication; in 332 complications, timing was established in 177 (53%) cases with 6 and 5 complications of E1 and E2, respectively. L1 and L2 were seen in 85 and 81 cases, respectively and no L3 complications were found. In 18 (8 of these were case reports) of the 41 studies, it was possible to connect the complication and the individual patient data. These 18 studies represent only 160 patients and we considered this number too low for subgroup analysis of complication risks. A few possible risk factors for complications could be estimated at a study group level. We found 31% complications per segment for the PRECICE nail and 46% complications per segment for the FITBONE nail. Surgical unit experience was assessed by dividing studies into studies with less than 20 patients (49% complications per segment) and studies with more than 40 patients (30% complications per segment) (see Supplementary data 6 for studies included in sub-analysis and Supplementary data 7 for full data presentation).

The inter-rater agreement between the 2 assessors of complications was 0.87 for severity grading and 0.94 for categorization of origin.

## Discussion

To our knowledge, this is the first systematic review on complications related to bone lengthening nails. The primary outcome was the risk of type IIIB complications resulting in a new pathology or permanent sequelae. This review found such IIIB complications in 3% of lengthened segments. Furthermore, a complication of any type was found in 34% of lengthened segments, and 5% of segments did not achieve the planned lengthening due to a complication (IIIA). In 15% of segments treated with intramedullary PRECICE and FITBONE lengthening nails, a complication (II) resulted in substantial change in treatment, such as unplanned re-surgery. 6% (11/177) of time-determined complications occurred intra- or perioperatively prior to start of distraction, and 94% of complications (166/177) occurred during or after the end of distraction. The high diversity of complications demonstrates that several means must be applied to reduce the high number of complications in intramedullary bone lengthening. Concerning the primary outcome, where the (type IIIB) complication resulted in a new pathology or permanent sequelae, the majority of complications were a result of joint-related complications such as contracture, subluxation, or dislocation. It is likely that a reduction in joint-related complications is accomplished by improved patient selection and attention to soft-tissue release as well as individualized protocols for lengthening, temporary extraarticular screw arthrodesis, splints/orthoses, or physiotherapy. The risk of joint subluxation and dislocation was 6 and 1 per 1,000 segments, respectively. Joint contracture was seen in 5% (53/983) of the segments, and primary soft-tissue release might be a key to address this complication; this was, however, only reported in 5 of the 41 studies (Shabtai et al. 2014, Paley et al. 2015, Laubscher et al. 2016, Rozbruch 2017, Calder et al. 2019). Calder et al. made a systematic division of the iliotibial band (ITB) if the planned lengthening was above 3 cm. They found that, in femoral lengthening, females lost joint movement in the hip and knee earlier than males. Moreover, it took substantially more time to regain range of motion in patients treated with retrograde compared with antegrade nails. However, we believe that higher rates of severe joint complications must be anticipated in high-risk patients such as congenital femoral deficiency and fibular hemimelia. We believe there is a need for systematic reporting of primary soft-tissue release as there is a lack of knowledge of benefits and challenges concerning this issue.

A device-related complication was seen in 12% (122/983) of segments with 5% (45/983) assigned to distraction mechanism-related complications, and 1% (13/983) of segments did not reach the lengthening goal due to device-related type IIIA complications. The overall complication rate per segment was 46% for studies only reporting the use of a FITBONE nail and 31% for studies only reporting the use of a PRECICE nail. However, the quality of data is not sufficient to compare complication rates between the 2 nail types; for example, did the studies that used only FITBONE nails include tibial lengthening in 27% compared with 16% in the PRECICE studies. In addition, in the FITBONE group the average number of patients per study was only 13 compared with an average number of 28 patients per study in the PRECICE group. However, the relatively high rate of device-related complications shown in this review warrants a constant focus on the technology of bone lengthening nails. Future studies should specifically report the type and generation of the applied nail to assess complication risk related to different nails and generations.

Complications related to bone regeneration were mainly due to delayed healing in 5% (46/983) of segments or premature consolidation in 2% (19/983) of segments. These complications might be reduced by increasing knowledge and handling of nail stability, patient compliance, mobilization, and biological factors such as type of osteotomy, latency period, and distraction rate/force. Another solution might be providing real-time feedback on surrogate markers of bone healing to allow for individualized distraction treatment. It seems logical that a surgeon’s ability to avoid or recognize, manage, and solve complications strongly correlates with the surgeon’s experience of this highly specialized treatment. This was to some extent supported by this review as studies with fewer than 20 patients had more complications per segment compared with studies with more than 40 patients.

The validity of a review depends on the quality of the included studies and on the validity of the data extraction. The level of evidence in the studies included in this review was low. Of the 41 included studies, there were 1 level 3 study, 32 level 4 studies, and 8 case reports and mean MINORS scores of about half of the ideal scores. Our study found 146 type II complications compared with 113 type I complications, and most patients thus had a more complex type of complication. This might reflect both underreporting and the lack of accurate reporting of complications in elective surgery (Martin et al. 2002).

We assessed complications in relation to segment lengthening and not to each patient because, in some patients, bone lengthening occurred in more than 1 bone in the same leg, lengthening involved both legs (short stature patients), and some patients underwent multiple lengthening procedures of the same bone. Complications per segment were lower than complications per patient, but since most of the patients had lengthening of only one segment, segment was chosen for main reporting.

41 studies with 983 bone lengthening segments reported either complications or stated absence of complications. With the increased popularity of lengthening by PRECICE and FITBONE nails, there is a knowledge gap concerning the distribution of severity grade and origin of complications in all treated patients. We believe that the demographics and number of included patients in this review are sufficiently diverse to illuminate even rare complications.

4 different classifications for reporting severity in bone lengthening complications were used (Paley 1990, Dahl et al. 1994, Dinçyürek et al. 2012, Black et al. 2015), and 29 studies did not use a classification. We are familiar with at least 4 more classifications of complications in limb lengthening, which challenges comparison between reported complications (Caton et al. 1985, Popkov 1991, Donnan et al. 2003, Lascombes et al. 2012). In this review we have classified the reported complications to achieve consistent reporting. However, it is a limitation that data on complications could be classified only from reported complications and not from original data. In the case of uncertainty between different grades of a complication, the complication was graded with the lower severity grade. Thereby, a systematic risk of reporting too low a complication severity grade was introduced. Another limitation of our review is that the reported complication rates could not be specified on subgroup level. We would expect that the complication rates differ substantially between a patient with idiopathic lower limb lengthening undergoing 3 cm of simple antegrade femoral lengthening without deformity correction and a patient with congenital fibular hemimelia and multiple previous operations undergoing 5 cm of tibial lengthening. However, it was not possible to extract data on a single patient level from the current literature. Therefore, we could not make correlations between complication rates and individual risk factors. We encourage future studies to report complications on a single patient level where complications can be related to possible risk factors such as age, diagnosis/etiology, segment, approach, nail type nail, nail generation, and timing of complications.

## Conclusion

This review of the literature shows an overall high rate of complications, with complications occurring in 1 of every 3 segments undergoing lower limb lengthening. In 1 of every 4 segments, complications have a major impact leading to substantial change in treatment (15%), failure to achieve lengthening goal (5%), or introduction of a new pathology or permanent sequelae (3%). As no standardized method of reporting complications exists, the true complication rate might be different. A standardized reporting method would substantially improve the knowledge needed to reduce the rate of complications.

## Supplementary Material

Supplemental Material
