# Supplementary material for: Systematic review of complications with externally controlled motorized intramedullary bone lengthening nails (FITBONE and PRECICE) in 983 segments
Source: Acta Orthop. 2020 Oct 27;92(1):120–7. doi: 10.1080/17453674.2020.1835321 (PMC7919879; doi:10.1080/17453674.2020.1835321)
Supplement: Supplemental Material [file IORT_A_1835321_SM6576.pdf]

Supplementary data

Supplementary data 1: Search strategy for the 3 electronic databases

PubMed

Search	Query
#11	Search ((((“Bone Lengthening”[Mesh]) OR bone lengthening*[Text Word]) OR leg lengthening*[Text Word])) AND (((((“Bone Nails”[Mesh]) OR (((magnetic*[Text Word] OR motorised[Text Word] OR motorized[Text Word])))) OR ((fitbone[Text Word] OR precice[Text Word]))) OR bone lengthening nail*[Text Word]) OR bone nail*[Text Word])
#10	Search ((((“Bone Nails”[Mesh]) OR (((magnetic*[Text Word] OR motorised[Text Word] OR motorized[Text Word])))) OR ((fitbone[Text Word] OR precice[Text Word]))) OR bone lengthening nail*[Text Word]) OR bone nail*[Text Word]
#9	Search bone nail*[Text Word]
#8	Search bone lengthening nail*[Text Word]
#7	Search (fitbone[Text Word] OR precice[Text Word])
#6	Search ((magnetic*[Text Word] OR motorised[Text Word] OR motorized[Text Word]))
#5	Search “Bone Nails”[Mesh]
#4	Search ((“Bone Lengthening”[Mesh]) OR bone lengthening*[Text Word]) OR leg lengthening*[Text Word]
#3	Search leg lengthening*[Text Word]
#2	Search bone lengthening*[Text Word]
#1	Search “Bone Lengthening”[Mesh]

Embase

No.	Query
#11	#4 AND #10
#10	#5 OR #6 OR #7 OR #8 OR #9
#9	‘bone nail*’
#8	‘lengthening nail*’
#7	fitbone OR precice
#6	magnetic* OR motorised OR motorized
#5	‘intramedullary nail’/exp
#4	#1 OR #2 OR #3
#3	‘bone lengthening*’
#2	‘leg lengthening*’
#1	‘leg lengthening’/de

Cochrane

ID	Search
#1	MeSH descriptor: [Bone Lengthening] explode all trees
#2	(bone NEXT lengthening):ti,ab,kw
#3	(leg NEXT lengthening):ti,ab,kw
#4	{OR #1-#3}
#5	MeSH descriptor: [Bone Nails] explode all trees
#6	(magnetic* OR motorised OR motorized):ti,ab,kw
#7	(fitbone OR precice):ti,ab,kw
#8	(lengthening NEXT nail*):ti,ab,kw
#9	(bone NEXT nail*):ti,ab,kw
#10	{OR #5-#9}
#11	#4 AND #10

Supplementary data 2

Disease etiology was grouped into 3 items: Congenital, Short stature, and Acquired/developmental limb-length discrepancy. Items were constructed by modification in accordance with Stricker and Hunt classification (Stricker and Hunt 2004)

**Congenital**
Congenital disease
Tibial hemimelia
Fibular hemimelia
Developmental coxa vara
Developmental dysplasia of the hip
Proximal femoral focal deficiency
Hemihypertrophy idiopathic
Nonsyndromic hemihypertrophy
Congenital tibial pseudarthrosis
Congenital posteromedial bowing tibia
Syndrome
Klippel–Trenaunay syndrome
Neurofibromatosis
Beckwith–Wiedemann syndrome
Ollier disease (multiple enchondromas)
Russell–Silver
Proteus
Conradi–Hunerman
Vivid cutis marmorata
Hemiatrophy
**Short stature**
Short stature cosmetic
Achondroplasia
Growth-hormone deficiency
**Acquired/developmental limb-length discrepancy**
Physeal growth disturbance
Ischemic physeal arrest (Perthes, post-infectious, limb ischemia, septic shock)
Blount’s disease (tibia vara)
Radiation therapy
Juxta-physeal tumor or bone cyst
Multiple exostosis/osteochondromatosis
Trauma
Traumatic physeal growth arrest
Fracture malunion (overriding)
Slipped capital femoral epiphysis (SCFE)
Hyperemia
Post-traumatic overgrowth (common after femur shaft fracture)
Chronic knee synovitis with overgrowth
Chronic osteomyelitis
Hemophilia
Rheumatoid arthritis
Osteoid osteoma
Arterio-venous malformation (AVM) or hemangiomatosis
Post-surgical hyperemia
Neuromuscular
Poliomyelitis
Spastic hemiplegia (cerebral palsy, stroke)
Spinal cord anomaly (tethered cord, syrinx)
Adult
Malunion
Post-traumatic and bone infection
Secondary to acute shortening
Non-union
Bone infection

Supplementary data 3

Complication was scored by time point using the following items.

Early complication
- E1. Intraoperative complication
- E2. Postoperative complication prior to distraction start

Late complication
- L1. Distraction period
- L2. After end of distraction and prior to implant removal
- L3. After implant removal

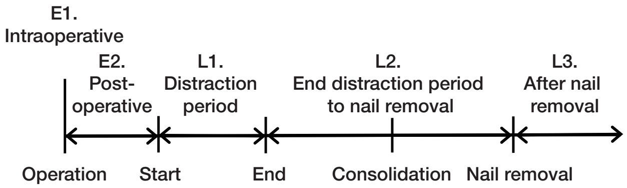

Supplementary data 4

Main origin	Sub-origin	Definition
Soft tissue	Skin	Skin irritation related to incision, internal/external devices, braces, or other treatment-related issues
	Muscles	Muscles irritation/pain/capturing/rupture related to incision, internal devices, other treatment-related issues
	Tendon	Tendon irritation/pain/captured/rupture related to incision, internal devices, other treatment-related issues
	Pain	Pain related to the treated extremity that is assessed to originate from the treatment
	Others	Other soft tissue complications that are not classified in above categories, including compartment syndrome
Joint	Pain	Pain related to the joint above or below the treated bone
	Contracture	Reduced joint range of motion compared with start of treatment
	Subluxation	A subluxation of a joint is where a connecting bone is partially out of the joint
	Dislocation	A dislocation of a joint is a complete separation of the joints
	Others	Other joint complications that are not classified in above categories
Vascular	Vascular damage	Blunt injury or penetrating injury to a blood vessel causing thrombosis, bleeding, or permanent vessel damage
	Deep vein thrombosis /Pulmonary embolism	Deep vein thrombosis refers to blood clots in large veins of lower limb Pulmonary embolism is a blockage of an artery in the lungs by a substance
	Hemorrhage/hematoma	A hemorrhage is blood escaping from the circulatory system from damaged blood vessels A hematoma is a localized bleeding outside of blood vessels
	Others	Other vascular complications not classified in above categories
Bone	Premature consolidation	The bone regenerated forms bone bridge between the two bone segments. The bridge stops lengthening and an intervention more than standard lengthening is needed
	Delayed healing	Non-union or slow consolidation of the bone regeneration
	Secondary malalignment	Occurrence of new bone malalignment
	Fracture	A partial or complete break in the continuity of the bone
	Others	Other bone complications not classified in above categories
Neurology	Paresthesia	An abnormal dermal sensation with no apparent physical cause and of transient time
	Paralysis	Loss of muscle function in one or more muscles and/or sensory disturbances in the affected area. Can be permanent or transient
	Others	Other neurological complications that are not classified in above categories
Infection	Superficial soft tissue	Clinical soft tissue infected above the facies
	Deep soft tissue	Clinical soft tissue infected below the facies
	Osteomyelitis	Infected bone marrow
	Others	Other infectious complications not classified in above categories
Device-related [a]	Distraction mechanism	Runaway, difficult to distract, non-distracting, non-functioning, and running back
	Mechanical strength	Nail/ring/bar bending or breakage. Rotational instability
	Attachment failure	Failure screw/wire/pins failure
	Other	Others device-related complications not classified in above categories. Could be corrosion, tissue reaction
Others	Patient	Patient-related complication that cannot be classified elsewhere
	Surgical	Surgical-related complication that cannot be classified elsewhere
	Others	All other complications that cannot be classified elsewhere

[a] (modified) (Lee et al. 2017)

Supplementary data 5

The following 3 quality assessment tools were used:

Quality assessment tool: METHODOLOGICAL INDEX FOR NON-RANDOMIZED STUDIES (MINORS) (Slim et al. 2003)

The items are scored 0 (not reported), 1 (reported but inadequate) or 2 (reported and adequate). The global ideal score is 16 for non-comparative studies and 24 for comparative studies.

MINORS—General part

1. ***A clearly stated aim:*** the question addressed should be precise and relevant in the light of available literature.
2. ***Inclusion of consecutive patients:*** all patients potentially fit for inclusion (satisfying the criteria for inclusion) have been included in the study during the study period (no exclusion or details about the reasons for exclusion).
3. ***Prospective collection of data:*** data were collected according to a protocol established before the beginning of the study.
4. ***Endpoints appropriate to the aim of the study:*** unambiguous explanation of the criteria used to evaluate the main outcome, which should be in accordance with the question addressed by the study. Also, the endpoints should be assessed on an intention-to-treat basis.
5. ***Unbiased assessment of the study endpoint:*** blind evaluation of objective endpoints and double-blind evaluation of subjective endpoints. Otherwise the reasons for not blinding should be stated.
6. ***Follow-up period appropriate to the aim of the study:*** the follow-up should be sufficiently long to allow the assessment of the main endpoint and possible adverse events.
7. ***Loss to follow-up less than 5%:*** all patients should be included in the follow-up. Otherwise, the proportion lost to follow-up should not exceed the proportion experiencing the major endpoint.
8. ***Prospective calculation of the study size:*** information on the size of detectable difference of interest with a calculation of 95% confidence interval, according to the expected incidence of the outcome event, and information about the level for statistical significance and estimates of power when comparing the outcomes.

Additional criteria in the case of comparative study

9. ***An adequate control group:*** having a gold standard diagnostic test or therapeutic intervention recognized as the optimal intervention according to the available published data.
10. ***Contemporary groups:*** control and studied group should be managed during the same time period (no historical comparison).
11. ***Baseline equivalence of groups:*** the groups should be similar regarding the criteria other than the studied endpoints. Absence of confounding factors that could bias the interpretation of the results.
12. ***Adequate statistical analyses:*** whether the statistics were in accordance with the type of study with calculation of confidence intervals or relative risk.

Quality assessment tool specifically concerning harm: McHarm scale from McMaster University (Santaguida et al. 2011, Kronick et al. 2014).

3 items from the McHarm scale were selected; items were scored as Yes or No.

1. Were the harms **predefined** using standardized or precise definitions?
2. Did the author(s) use **standard** scale(s) or checklist(s) for harms collection?
3. Did the author(s) specify the **number** for each **type** of harmful event for each study group?

Quality assessment tool for case report: Methodological quality and synthesis of case series and case reports (Murad et al. 2018)

Items were scored as Yes (1) or No (0). The global ideal score was 8.

1. Does the patient(s) represent(s) the whole experience of the investigator (center) or is the selection method unclear to the extent that other patients with similar presentation may not have been reported?
2. Was the exposure adequately ascertained?
3. Was the outcome adequately ascertained?
4. Were other alternative causes that may explain the observation ruled out?
5. Was there a challenge/rechallenge phenomenon?
6. Was there a dose–response effect?
7. Was follow-up long enough for outcomes to occur?
8. Is the case(s) described with sufficient details to allow other investigators to replicate the research or to allow practitioners make inferences related to their own practice?

 Acta Orthopaedica 2020; 91 (DOI: 10.1080/17453674.2020.1835321)

Supplementary data 6

Data from NON-RANDOMIZED STUDIES without comparative part 1

Study reference [a]	A	B	C	D	E	F	G	H	I
Level of evidence	IV	IV	IV	IV	IV	IV	IV	IV	IV
Included in sub-analysis [b]	F / 20	F	F / 20	F / 20	P	P / 20	P	F / 20	F
MINORS Quality assessment tool									
1. A clearly stated aim	2	2	2	2	2	2	1	2	1
2. Inclusion of consecutive patients	2	1	1	0	2	0	0	0	2
3. Prospective collection of data	2	2	2	0	0	0	0	2	0
4. Endpoints appropriate to the aim of the study	2	2	2	2	1	2	1	2	2
5. Unbiased assessment of the study endpoints	0	0	0	0	0	0	0	0	0
6. Follow-up period appropriate to the aim of the study	2	2	2	2	0	2	1	2	2
7. Loss to follow-up less than 5%	2	2	2	2	2	2	2	2	2
8. Prospective calculation of the study size	0	0	0	0	0	0	0	0	0
Sum	12	11	11	8	7	8	5	10	9
Assessing quality of harms assessment (McMaster selected questions): Yes/no									
1. Were the harms PREDEFINED using standardized or precise definitions?	No	No	No	No	No	No	No	No	No
2. Did the author(s) use STANDARD scale(s) or checklist(s) for harms collection?	Yes	Yes	No	Yes	No	No	No	No	Yes
3. Did the author(s) specify the NUMBER for each TYPE of harmful event for each study group?	Yes	Yes	Yes	Yes	Yes	Yes	No	Yes	Yes

[a] Study references: A. (Accadbled et al. 2019); B. (Accadbled et al. 2016); C. (Al-Sayyad 2012); D. (Dinçyürek et al. 2012); E. (Haider and Wozasek 2019); F. (Hammouda et al. 2017); G. (Karakoyun et al. 2016); H. (Krieg et al. 2008); I. (Krieg et al. 2011)
[b] F = FITBONE; P = PRECICE

Data from NON-RANDOMIZED STUDIES without comparative part 2

Study reference [a]	A	B	C	D	E	F	G	H	I
Level of evidence	IV	IV	IV	IV	IV	IV	IV	IV	IV
Included in sub-analysis [b]	P	P	F	F / 20	P / 20	P / 20	F / 20	P / 40	P
MINORS Quality assessment tool									
1. A clearly stated aim	2	2	1	1	2	2	2	2	2
2. Inclusion of consecutive patients	2	0	0	2	0	2	2	2	0
3. Prospective collection of data	2	2	0	0	0	0	0	0	0
4. Endpoints appropriate to the aim of the study	2	2	2	2	1	2	2	2	2
5. Unbiased assessment of the study endpoints	0	0	0	0	0	0	0	0	0
6. Follow-up period appropriate to the aim of the study	1	1	2	2	2	1	2	2	2
7. Loss to follow-up less than 5%	0	0	2	2	2	0	2	0	2
8. Prospective calculation of the study size	0	0	0	0	0	0	0	0	0
Sum	9	7	7	9	7	7	10	8	8
Assessing quality of harms assessment (McMaster selected questions): Yes/no									
1. Were the harms PREDEFINED using standardized or precise definitions?	No	No	No	No	No	No	No	No	No
2. Did the author(s) use STANDARD scale(s) or checklist(s) for harms collection?	Yes	No	No	No	Yes	Yes	Yes	Yes	No
3. Did the author(s) specify the NUMBER for each TYPE of harmful event for each study group?	Yes	Yes	Yes	Yes	Yes	Yes	Yes	Yes	Yes

[a] Study references: A. (Schiedel et al. 2014); B. (Shabtai et al. 2014); C. (Singh et al. 2006); D. (Steiger et al. 2018); E. (Tiefenboeck et al. 2016); F. (Wiebking et al. 2016); G. (Lenze et al. 2011); H. (Frommer et al. 2018); I. (Kirane et al. 2014)
[b] F = FITBONE; P = PRECICE

Data from NON-RANDOMIZED STUDIES without comparative part 3

Study reference [a]	A	B	C	D	E	F	G	H
Level of evidence	IV	IV	IV	IV	IV	IV	IV	IV
Included in sub-analysis [b]	P	P / 20	F / 20	P	20	P	F	P / 40
MINORS Quality assessment tool								
1. A clearly stated aim	2	2	1	2	1	2	1	1
2. Inclusion of consecutive patients	1	2	0	2	0	2	0	1
3. Prospective collection of data	0	0	0	0	0	2	0	0
4. Endpoints appropriate to the aim of the study	2	2	1	2	2	2	2	2
5. Unbiased assessment of the study endpoints	0	0	0	0	0	0	0	0
6. Follow-up period appropriate to the aim of the study	1	1	2	1	2	2	2	2
7. Loss to follow-up less than 5%	0	2	2	2	2	2	0	2
8. Prospective calculation of the study size	0	0	0	0	0	0	0	0
Sum	6	9	6	9	7	12	5	8
Assessing quality of harms assessment (McMaster selected questions): Yes/no								
1. Were the harms PREDEFINED using standardized or or precise definitions?	No	No	No	No	No	No	No	No
2. Did the author(s) use STANDARD scale(s) or checklist(s) for harms collection?	No	Yes	No	No	No	Yes	No	No
3. Did the author(s) specify the NUMBER for each TYPE of harmful event for each study group?	Yes	Yes	Yes	Yes	Yes	Yes	No	Yes

[a] Study references: A. (Iobst et al. 2018); B. (Birkholtz and De-Lange 2016); C. (Baumgart et al. 1997); D. (Cosic and Edwards 2020); E. (Havitcioglu et al. 2020); F. (Nasto et al. 2020); G. (Küçükkaya et al. 2015); H. (Paley et al. 2014)
[b] F = FITBONE; P = PRECICE

Data from NON-RANDOMIZED STUDIES with a comparative part

Study reference [a]	A	B	C	D	E	F	G
Level of evidence	III	IV	IV	IV	IV	IV	IV
Included in sub-analysis [b]	F / 20	P / 40		P	P / 40	P / 40	40
MINORS Quality assessment tool							
1. A clearly stated aim	2	2	2	2	2	2	2
2. Inclusion of consecutive patients	2	2	0	2	2	2	2
3. Prospective collection of data	0	0	0	0	0	0	0
4. Endpoints appropriate to the aim of the study	2	2	2	2	2	2	2
5. Unbiased assessment of the study endpoints	0	0	0	0	2	0	0
6. Follow-up period appropriate to the aim of the study	2	1	2	1	2	0	2
7. Loss to follow-up less than 5%	2	2	2	2	0	0	2
8. Prospective calculation of the study size	0	0	0	0	0	0	0
Additional criteria in the case of comparative study							
9. An adequate control group	2	2	2	2	2	2	2
10. Contemporary groups	2	2	2	0	0	0	2
11. Baseline equivalence of groups	1	1	2	0	0	2	2
12. Adequate statistical analyses	2	2	2	2	2	2	2
Sum	17	16	16	13	14	12	18
Assessing quality of harms assessment (McMaster selected questions): Yes/no							
1. Were the harms PREDEFINED using standardized or precise definitions?	No	No	No	No	No	No	No
2. Did the author(s) use STANDARD scale(s) or checklist(s) for harms collection?	Yes	No	No	No	Yes	Yes	Yes
3. Did the author(s) specify the NUMBER for each TYPE of harmful event for each study group?	Yes	Yes	Yes	Yes	Yes	Yes	Yes

[a] Study references: A. (Black et al. 2015); B. (Calder et al. 2019); C. (Karakoyun et al. 2015); D. (Laubscher et al. 2016); E. (Lee et al. 2017); F. (Paley et al. 2015); G. (Horn et al. 2019)
[b] F = FITBONE; P = PRECICE

 Acta Orthopaedica 2020; 91 (DOI: 10.1080/17453674.2020.1835321)

Data from case reports

First authors<br>Publication year	Couto<br>2018	Morrison<br>2016	Harkin<br>2018	Wu<br>2018	Muratori<br>2018	Baumgart<br>2005	Rozbruch<br>2017	Kariksiz<br>2019
Included in sub-analysis [b]	P / 20	P / 20	P / 20	P / 20	P / 20	F / 20	P / 20	P / 20
Quality assessment [c]								
1	1	1	0	1	0	1	0	1
2	1	1	1	1	1	1	1	1
3	1	1	1	1	1	1	1	1
4	0	1	0	0	0	0	0	0
5	0	0	0	0	0	0	0	0
6	0	0	0	0	0	0	0	0
7	1	1	1	0	1	0	1	1
8	1	1	1	0	0	1	1	1
Sum	5	6	4	3	3	4	4	5

[a] Study reference: (Couto et al. 2018), (Morrison and Sontich 2016), (Harkin et al. 2018), (Wu and Kuhn 2018), (Muratori et al. 2018), (Baumgart et al. 2005), (Rozbruch 2017), (Kariksiz and Karakoyun 2019)
[b] F = FITBONE; P = PRECICE
[c] Case reports Quality assessment tool by Murad et al. (Yes = 1, No = 0)

Supplementary data 7

Data from analysis of subgroups

FITBONE complications. Studies that only report use of a FITBONE nail were included. The included studies can be identified in Electronic Supplementary data 6 under included in sub-analysis, marked with FITBONE

Numbers of studies				13	
Numbers of segments				196	
Number of patients				165	
Age range				11–53	
Sex: M / F				63 / 47	
Unidentified regarding sex				55	
Congenital disease				48	
Short stature				13	
Acquired/developmental LLD				81	
Unidentified disease etiology				23	
Femur				144	
Tibia				52	
Severity grade of complications:	I	II	IIIA	IIIB	Sum
Number of complications	38	33	9	10	90
Complications per segment, %	19	17	5	5	46
Complications per patient, %	23	20	5	6	55

PRECICE complications. Studies that only report use of a PRECICE nail were included. The included studies can be identified in Electronic Supplementary data 6 under sub-analysis, marked with PRECICE

Numbers of studies				25	
Numbers of segments				699	
Number of patients				540	
Age range				8–74	
Sex: M / F				283 / 148	
Unidentified regarding sex				109	
Congenital disease				130	
Short stature				84	
Acquired/developmental LLD				191	
Unidentified disease etiology				135	
Femur				589	
Tibia				110	
Severity grade of complications:	I	II	IIIA	IIIB	Sum
Number of complications	73	102	29	16	220
Complications per segment, %	10	15	4	2	31
Complications per patient, %	14	19	5	3	41

Small case-series versus large case-series

As an indirect measure of experience, we have divided the studies into studies with less than 20 patients and studies with more than 40 patients. We have made the assumption that a higher number of patients reflect a higher volume and not just a longer inclusion period. We collected the studies into two groups. Group 1: Studies reporting less than 20 cases. Group 2: Studies reporting more than 40 cases. The studies including between 20 and 40 cases were not included.

Less than 20 patients per study. Studies that only report on fewer than 20 patients were included. The included studies can be identified in Electronic Supplementary data 6 under sub-analysis, marked with 20

Numbers of studies				21	
Numbers of segments				166	
Number of patients				144	
Age range				9–74	
Sex: M / F				86 /58	
Unidentified regarding sex				0	
Congenital disease				36	
Short stature				14	
Acquired/developmental LLD				94	
Unidentified disease etiology				0	
Femur				133	
Tibia				33	
Severity grade of complications:	I	II	IIIA	IIIB	Sum
Number of complications	27	35	11	8	81
Complications per segment, %	16	21	7	5	49
Complications per patient, %	19	24	8	6	56

More than 40 patients per study. Studies that only report on more than 40 patients were included. The included studies can be identified in Electronic Supplementary data 6 under sub-analysis, marked with 40

Numbers of studies				6	
Numbers of segments				475	
Number of patients				331	
Age range				9–68	
Sex: M / F				188 / 96	
Unidentified regarding sex				47	
Congenital disease				104	
Short stature				80	
Acquired/developmental LLD				106	
Unidentified disease etiology				41	
Femur				413	
Tibia				62	
Severity grade of complications:	I	II	IIIA	IIIB	Sum
Number of complications	39	67	26	9	141
Complications per segment, %	8	14	5	2	30
Complications per patient, %	12	20	8	3	43
